# A point-of-care thoracic ultrasound protocol for hospital medical emergency teams (METUS) improves diagnostic accuracy

**DOI:** 10.1186/s13089-021-00229-3

**Published:** 2021-06-04

**Authors:** M. J Blans, E Bousie, J. G van der Hoeven, F. H Bosch

**Affiliations:** 1 Department of Intensive Care, Rijnstate Hospital, PO box 9555, 6800 TA Arnhem, The Netherlands; 2 Department of Intensive Care, Radboud University Medical Center, PO box 9101, 6500 HB Nijmegen, The Netherlands; 3 Department of Internal Medicine, Rijnstate Hospital, PO box 9555, 6800 TA Arnhem, The Netherlands

## Abstract

**Background:**

Point-of-care ultrasound (POCUS) has proven itself in many clinical situations. Few data on the use of POCUS during Medical Emergency Team (MET) calls exist. In this study, we hypothesized that the use of POCUS would increase the number of correct diagnosis made by the MET and increase MET’s certainty.

**Methods:**

Single-center prospective observational study on adult patients in need for MET assistance. Patients were included in blocks (weeks). During even weeks, the MET physician performed a clinical assessment and registered an initial diagnosis. Subsequently, the POCUS protocol was performed and a second diagnosis was registered (US+). During uneven weeks, no POCUS was performed (US−). A blinded expert reviewed the charts for a final diagnosis. The number of correct diagnoses was compared to the final diagnosis between both groups. Physician’s certainty, mortality and possible differences in first treatment were also evaluated.

**Results:**

We included 100 patients: 52 in the US + and 48 in the US−  group. There were significantly more correct diagnoses in the US+ group compared to the US− group: 78 vs 51% (*P*  = 0.006). Certainty improved significantly with POCUS (*P*  <  0.001). No differences in 28-day mortality and first treatment were found.

**Conclusions:**

The use of thoracic POCUS during MET calls leads to better diagnosis and increases certainty.

*Trial registration*. ClinicalTrials.gov. Registered 12 July 2017, NCT03214809 https://www.clinicaltrials.gov/ct2/show/NCT03214809?term=metus&cntry=NL&draw=2&rank=1

**Supplementary Information:**

The online version contains supplementary material available at 10.1186/s13089-021-00229-3.

## Background

Medical Emergency Teams (METs) are called to the bedside when patients on hospital wards deteriorate. [1, 2] METs use various algorithms to assess the patient’s condition, most frequently the ABCDE method. The MET physician will use a combination of history, physical examination and point-of-care laboratory tests to assess the patient. The addition of point-of-care ultrasound (POCUS) could potentially improve diagnostic accuracy. [3] METs are associated with a reduction in patient mortality [4], but few data exist on differences in operation procedures by METs. The role of POCUS for instance during MET calls has not been investigated extensively yet, even though its role in the emergency room (ER) and intensive care unit (ICU) is well established [5–13]. Recently, Zieleskiewicz et al. published the first prospective observational study on the effect of the use of a multi-organ POCUS protocol during MET calls [14]. In this study, the use of a multi-organ POCUS protocol improved diagnostic accuracy of the MET significantly. We also designed a prospective trial hypothesizing that multi-organ POCUS would increase MET diagnostic ability and postulated that the use of POCUS would also increase the diagnostic certainty of the MET physician. Influence on certainty by the use of POCUS has been found in a study on ER patients [5] and improving certainty might also be important to attending MET physicians.

Because most MET calls are requested for respiratory and or hemodynamic deterioration [15], we designed a POCUS protocol consisting of cardiac and lung (thoracic) ultrasound.

## Methods

### Study design and setting

#### Design

This is a prospective observational study examining the use of thoracic POCUS in adult patients on the general ward treated by the MET. The Modified Early Warning Score (MEWS) was used to assess the need for MET assistance (figure of MEWS score in Additional file 1).

The study (METUS NL61884.091.17) was approved by the local ethical committee and conducted in a Dutch 750 bed teaching hospital (Rijnstate Hospital, Arnhem) from January 18, 2019 until February 1, 2020. The study was registered at ClinicalTrials.gov. (NCT03214809).

#### Characteristics of participants

All patients 18 years and older in all regular hospital wards in need of a MET call were included.

Exclusion criteria were:

- Pregnancy
- Acute illness requiring direct lifesaving intervention (e.g., intubation, cardiopulmonary resuscitation).
- Glasgow Coma Score < 9 or a decline of the Glasgow Coma Score ≥ 2 as the primary reason for MET attendance.

Patients consent was obtained directly after the MET call; in case of an incapacitated patient, the next of kin was contacted. Deferred consent was also permitted.

#### MET team staffing

The ICU of Rijnstate Hospital runs a MET since 1996. The MET is staffed by 2 intensive care nurses and 1 ICU resident physician. Board certified intensivists are available within 15 min. The ICU of Rijnstate Hospital uses POCUS since 2009. ICU residents are trained in basic POCUS shortly before ICU rotation. The training program consists of 4 training days in basic cardiac, lung and abdominal ultrasound, POCUS is part of daily care [16].

#### POCUS protocol

Our cardiac POCUS protocol consists of 5 straightforward questions combined with a simple qualitative interpretation. Standard transthoracic windows using only 2D-ultrasound were used [17].

The following questions were answered:

1. Is the left ventricle dilated?—yes/no/don’t know. Is the left ventricle function hyperdynamic/normal/moderately decreased/severely decreased/don’t know?
2. Is the right ventricle dilated?—yes/no/don’t know
3. Is the right ventricle function normal/abnormal/don’t know?
4. Is pericardial effusion present?—yes/no/don’t know
5. Is pericardial tamponade present?—yes/no/don’t know

From the subcostal view the inferior vena cava (IVC) was identified. The IVC was measured and categorized:

- Collapsed: < 1.5 cm.
- Normal: 1.5–2.5 cm.
- Dilated: > 2.5 cm.
- Not visualized.

Lung ultrasound was used according to the Blue protocol by Lichtenstein [18] with the following diagnostic profiles:

- A-profile: normal lung.
- A/A’-profile (one sided): suspect pneumothorax, atelectasis, pleurodesis, pneumonectomy.
- B-profile: (both sides) suspect pulmonary edema, acute respiratory distress syndrome (ARDS).
- A/B-profile (one sided B-lines): suspect pneumonia.
- C-profile (consolidation): suspect pneumonia, atelectasis or compression.

The MET physicians started with cardiac POCUS in case of primary hemodynamic problems and with the Blue protocol of the lungs in case of primary pulmonary problems.

We used a hand held ultrasound device (Philips Lumify® S4-1) connected to an Android tablet attached to the MET cart. The Lumify^R^ S4-1 is a phased array transducer with software for cardiac and lung ultrasound exams.

### Data collection

Eligible patients were included consecutively: in even weeks the POCUS protocol was used (US+), in odd weeks standard care without the use of POCUS (US−) was deployed.

After the initial assessment, a diagnosis was registered by the MET physician.

In the US+ weeks, a second diagnosis was registered after subsequent use of the POCUS protocol. The attending MET physician could decide to use POCUS in the US- weeks after an initial diagnosis was made. This deviation of protocol was registered and, in these cases, also a second diagnosis (after the use of POCUS) was noted.

All diagnoses were recorded in a case research form (CRF).

An experienced intensive care consultant and member of the hospital mortality committee (independent expert) conducted a full chart review (electronical medical record, HIX®) on all enrolled patients to determine a definite diagnosis 2 weeks after inclusion. The independent expert was unaware of the ultrasound findings (recorded in a separate CRF), the initial diagnosis made by the attending MET physician and he had no other role in the study. After evaluation by the independent expert, the MET diagnosis was rated as completely correct or completely incorrect. In case of multiple definite diagnosis made by the independent expert (for instance, acute heart failure and COPD), the MET diagnosis could also be rated partially correct if not all elements of the definite diagnosis were recorded in the CRF.

Diagnostic certainty was scored on a visual analogue scale of 0 (no clue) to 10 (absolute certain). The 10 point VAS scale was used because all other clinical scoring in our hospital is done with the 10 points VAS score (for instance pain). Other scales like the 5-point Likert scale would be novel for our physicians to use thereby possibly clouding the results. Ten point VAS scores have been used in other studies before in certainty assessment [19]. In the US+ weeks the MET physician rated certainty before and after the use of POCUS. In the US− weeks certainty was scored without the use of POCUS and in case of protocol deviation also after the use of POCUS.

We also registered the reason the MET was called, baseline demographics (age, gender, previous medical history, weight and height), clinical and laboratory parameters (heart rate, blood pressure, temperature, serum lactate and white blood cell count) and 28-day mortality.

The MET physicians were asked to rate the quality of the POCUS studies (good, moderate, bad) and were encouraged to capture the POCUS studies for review. Two investigators (FHB and MJB) checked the stored studies.

### Outcome measures

The primary outcome measure was the percentage of correct diagnoses made by the MET physician in the US+ and US− weeks. The second diagnosis after the use of POCUS in the US+ weeks and the initial diagnosis in the US− weeks without the use of POCUS were compared to the final diagnosis made by the independent expert.

Secondary outcome measures were a change in diagnosis after the use of POCUS in the US+ group and (after protocol deviation) in the US− group, percentage of correct diagnosis in the US− group after the use of POCUS, the change in diagnostic certainty before and after the use of POCUS and 28-day mortality. The MET physician also noted first treatment (intravenous fluids, diuretics, vasopressors/inotropes, anti-coagulants, anti-arrhythmic drugs, vasodilators, morphine/sedatives, intubation or non-invasive ventilation, O_2_ supply, or other treatments and the need for supervisor attendance).

### Statistical analysis

Descriptive statistics are presented as mean with standard deviation for normally distributed continuous data, median and inter-quartile range (IQR) for skewed continuous variables and as numbers and percentages for dichotomous and categorical variables. Differences between groups (US+ group and US− group) were tested using the Pearson Chi-square test, Fisher exact and Students’ *T* test. In case of not normally distributed variables, differences between groups were tested using the Mann–Whitney *U* test. Changes within groups were tested using the Wilcoxon signed rank test and the McNemar–Bowker test. Statistical analysis was done using SPSS® software (version 25). Sample size was estimated to detect an increase in the number of correct diagnosis of 30% (*α* = 0.05 and *β* = 0.20). Based on Jones et al. [13] we estimated that a total of 76 patients should be enrolled (38 patients per group). Because few data exist on the use of POCUS during MET calls we decided to include 100 patients in total.

## Results

We included a total of 100 patients, 52 patients in the US+ group and 48 patients in the US− group. In 5 patients the independent expert could not determine a reliable definite diagnosis (2 in the US+ and 3 in the US− group). These patients were excluded from the comparison of initial/second diagnosis with the definite diagnosis, but were included in other analysis. In total, there were 310 MET calls during the study period.

**Fig. 1:**
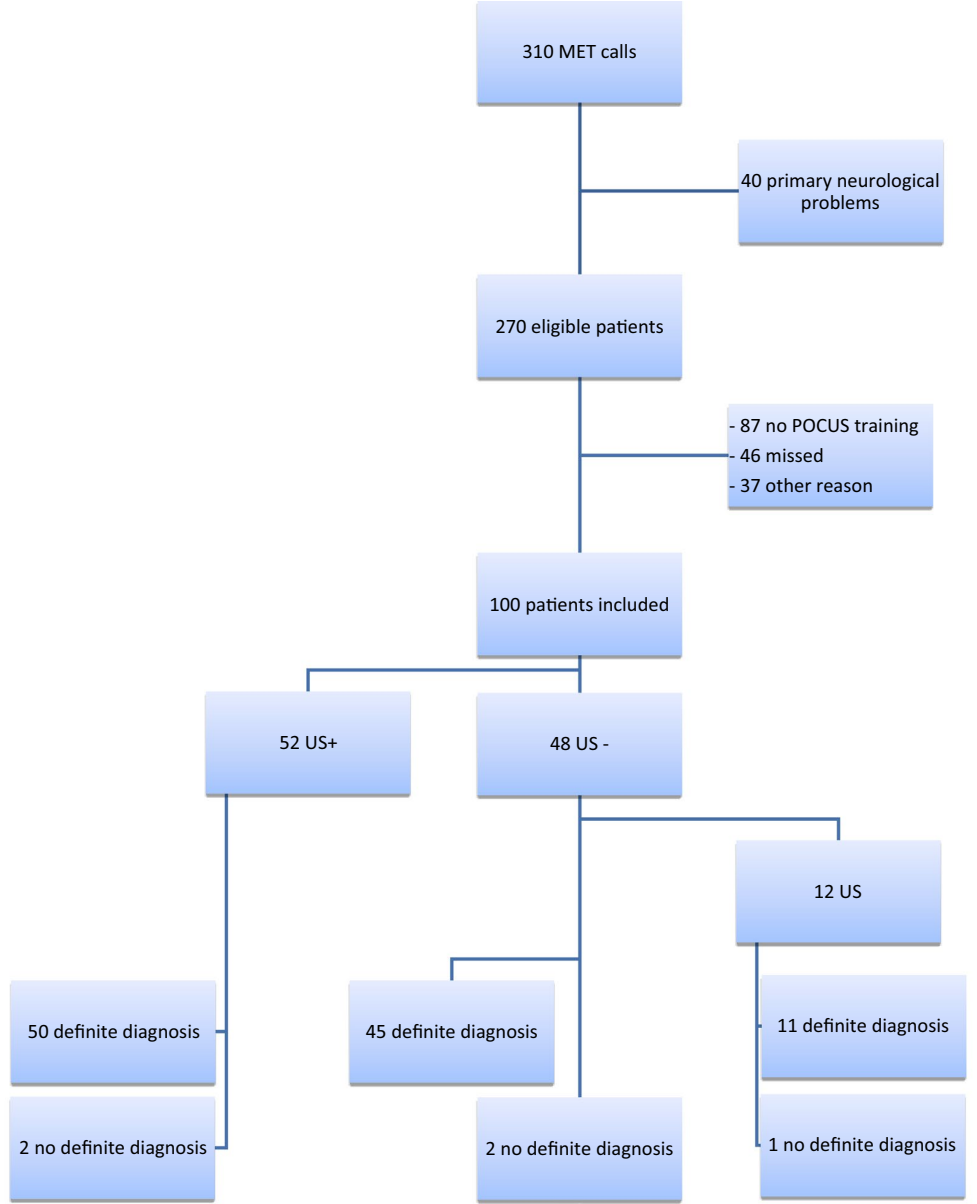
Flowchart of patient study enrollment

Flowchart of study enrollment and exclusion reasons are in listed in Fig. 1.

Patients characteristics are described in Table 1.

**Table 1:** Baseline characteristics

	Even week		Uneven week		*P* value
	N = 52		N = 48		
	Mean (SD)	N (%)	Mean (SD)	N (%)	
Age (years)	72.2 (15.0)		69.7 (12.1)		0.354
Gender, male		28 (53.8)		27 (56.3)	0.803
BMI^a^ (kg/m^2^)	26.5 (5.9)		28.2 (5.8)		0.175
Systolic BP (mmHg)	134 (36.5)		127 (42.9)		0.408
Diastolic BP (mmHg)	78 (22.0)		76 (26.2)		0.544
Heart rate (bpm)	110 (33.4)		111 (32.9)		0.870
Temperature (°C)	37.5 (1.6)		37.5 (1.2)		0.859
WBC^b^ (× 10^9^/L)	10.4 (5.2)		11.5 (7.6)		0.491
Plasma lactate (mmol/L)	3.1 (2.6)		3.3 (2.6)		0.731
Reason for call					
Anaphylaxis		0 (0.0)		1 (2.1)	0.296
Gastro-intestinal bleeding		2 (3.8)		0 (0.0)	0.175
Hypotension		16 (30.6)		18 (37.5)	0.469
Respiratory insufficiency		34 (65.4)		27 (56.2)	0.349
Tachycardia		0 (0.0)		2 (4.2)	0.137
Pre-existing condition					
Heart failure		14 (26.9)		5 (10.4)	0.037*
Myocardial infarction		12 (23.1)		4 (8.3)	0.045*
Peripheral vascular		9 (17.3)		5 (10.4)	0.323
COPD^c^		14 (26.9)		13 (27.1)	0.982
Renal insufficiency		16 (30.8)		7 (14.6)	0.056
Dialysis		1 (1.9)		1 (2.1)	0.943
Diabetes mellitus		8 (15.4)		8 (16.7)	0.860
Metastatic malignancy		4 (7.8)		7 (14.6)	0.281
Immunological insufficiency		1 (1.9)		2 (4.2)	0.503
Gastro-intestinal bleeding		2 (3.8)		0 (0.0)	0.175
Haematological malignancy		0 (0.0)		2 (4.2)	0.137

*P < 0.05

^a^BMI body mass index

^b^WBC white blood cells

^c^COPD chronic obstructive pulmonary disease

### Primary outcome

#### Percentage of correct diagnosis US+ versus US− group

In the US+ group 39 (78%) of the diagnoses after the use of POCUS was completely correct versus 23 (51.1%) of the diagnosis without the use of POCUS in the US− group (Pearson Chi Square Test: *P* = 0.006) (Table 2.)

**Table 2:** Initial diagnosis versus final diagnosis US + and US

Initial diagnosis compared to final diagnosis	US+ weeks Number (%)	US− weeks Number (%)	Total Number (%)
Completely correct	39 (78.0)	23 (51.1)	62 (65.3)
Partly correct	8 (16.0)	9 (20.0)	17 (17.9)
Completely incorrect	3 (6.0)	13 (28.9)	16 (16.8)
Total	50 (100)	45 (100)	95 (100)

### Secondary outcomes

#### Change in diagnosis

In the US+ group, the initial diagnosis improved to partly correct in 3 (6%) and to completely correct in 10 (20%) of the patients (McNemar–Bowker test P = 0.004). In 3 (6%) patients an incorrect diagnosis was not improved with the use of POCUS and in 3 (6%) patients the diagnosis after ultrasound worsened from completely correct to partially correct. In no case the diagnosis changed from completely correct before to completely incorrect after the use of POCUS (Table 3).

**Table 3:** Initial versus diagnosis after POCUS in the US + group

Initial diagnosis before POCUS				
	Completely correct Number (%)	Partly correct Number (%)	Completely incorrect Number (%)	Total Number (%)
Diagnosis after POCUS				
Completely correct	26 (52)	3 (6.0)	10 (20.0)	39 (78.0)
Partially correct	1 (2.0)	7 (14.0)	0 (0.0)	8 (16.0)
Completely incorrect	0 (0.0)	0 (0.0)	3 (6.0)	3 (6.0)
Total	26 (53.1)	10 (20.0)	13 (26.0)	50 (100)

#### Percentage of correct diagnosis in the US- group after the use of POCUS

In 12 (25%) patients in the US- group there was a protocol deviation. In 11 of these patients, a final diagnosis could be established. POCUS increased the agreement with the final diagnosis from 27.3% to 63.6%, but this difference was not statistically significant, due to the small sample size.

The actual diagnoses before and after POCUS are listed in Table 4.

**Table 4:** List of diagnosis before and after ultrasound in the US+ and US− group

US+	
Diagnosis before ultrasound (completely incorrect)	Diagnosis after ultrasound (completely correct)
No diagnosis	Hypoventilation
No diagnosis	Respiratory problems due to abdominal disease
No diagnosis	Acute heart failure
Pneumonia	Acute heart failure + pneumonia
Acute heart failure	Pneumonia + septic shock
Acute pulmonary embolism	Hypoventilation
Acute heart failure	Underfilling
Acute exacerbation COPD	Pulmonary Fibrosis
Acute heart failure	Pneumonia
Acute exacerbation COPD	Acute heart failure

Diagnosis before ultrasound (partially correct)	Diagnosis after ultrasound (completely correct)
Pneumonia + atelectasis	Atelectasis
Acute exacerbation COPD	Acute heart failure + exacerbation COPD
Retention bladder	Septic shock + retention bladder

US −	
Diagnosis before ultrasound (completely incorrect)	Diagnosis after ultrasound (completely correct)
Pneumonia + exacerbation ILD	Acute heart failure
Tension pneumothorax	Haemothorax
Tension pneumothorax	Atelectasis

Diagnosis before ultrasound (partially correct)	Diagnosis after ultrasound (completely correct)
Septic shock	ARDS

*COPD* chronic obstructive pulmonary disease, *ILD* interstitial lung disease, *ARDS* acute respiratory distress syndrome

#### Certainty

**Fig. 2:**
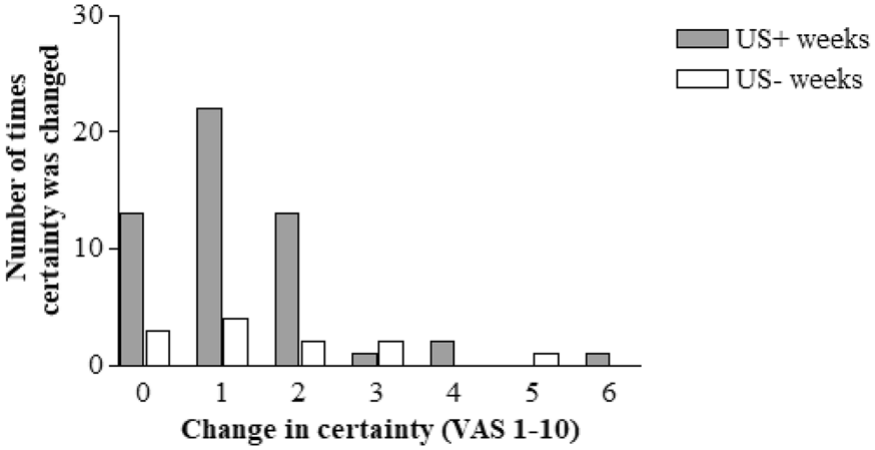
Difference in certainty before and after US in US− weeks after the use of POCUS

Diagnostic certainty before the use of POCUS was the same in the US + and US− groups. In both groups the median certainty was 8 with comparable ranges. Certainty improved in the US+ group after the use of POCUS (Wilcoxon signed rank test *P* < 0.001) (Fig. 2).

This was also found in the 12 patients from the US- group in which ultrasound was used (Fig. 2) Wilcoxon signed rank test *P* = 0.001. In the minority of cases, the use of POCUS did not increase certainty.

#### Mortality

28-Day mortality rates were not statistically different: US+ weeks 14 (26.9%) and the US− weeks 13 (27.1%).

#### Initial treatment and need for immediate supervisor attendance

No statistically significant differences were found between the US+ and US− groups in first treatment (intravenous fluids, diuretics, vasopressors/inotropes, anti-coagulants, anti-arrhythmic drugs, vasodilators, morphine/sedatives, intubation or non-invasive ventilation, O_2_ supply, or other treatments) nor in the number of times supervisor attendance was needed.

#### Quality of POCUS exams

The MET physicians rated the quality of ultrasound exams as good in 25 (39%), moderate 27 (42%) and poor in 12 (19%). One in five studies were evaluated by two experts (FHB and MJB), in one case the quality was adjusted from good to moderate, in all other cases the experts agreed on the rating of POCUS study quality done by the MET physician.

## Discussion

In this single-center prospective observational study the use of a thoracic POCUS protocol improved the number of correct diagnosis significantly. We also found that the number of correct diagnosis increased in the US+ group after the use of POCUS and that POCUS increased MET physician’s certainty significantly. We did not find differences in mortality, first MET treatments or supervisor attendance.

There are multiple studies evaluating the use of POCUS in the ICU and ER department, until now only two studies focused on the use of POCUS during MET calls. Zieleskiewicz et al. published a paper [14] in which they evaluated the effect on diagnostic adequacy of thoracic POCUS during MET calls. As in our study, they found a significant increase in the number of correct diagnosis when POCUS was used (80% versus 94%). Furthermore, the time to first treatment was significantly lower in the POCUS group and there was an association with outcome parameters such as mortality, but the latter was not confirmed in the propensity score. Although this study has many similarities with ours, there are also some important differences. Both studies are single centered and prospective observational studies. The inclusion criteria are comparable as is the inclusion rate over time and the number of inclusions out of the total MET calls (34% Zieleskiewicz et al. versus 32% Blans et al.). In both studies, the protocol consisted of cardiac and lung POCUS, Zieleskiewicz et al. also used vascular POCUS to rule out lower extremity thrombosis, but the latter was used infrequently. Both studies found that the use of POCUS during MET calls improved the number of correct diagnosis made by the MET physician (primary endpoint). In both studies, chart review was used to establish a definite diagnosis, but there are some differences in the exact way in which this process was carried out. In contrast to the Zieleskiewicz et al. study, we used the term “partly” correct diagnosis if not all elements of the definite diagnosis made by the external expert were scored by the MET physician. If we add the partly correct to the completely correct numbers, our results would increasingly be comparable to the Zieleskiewicz results (US+ group 94% correct and US− group 69% correct).

Another important methodological difference with our study is the fact that Zieleskiewicz et al. used two MET teams, one which used POCUS and one did not. The two MET’s alternated every other day. Both MET deployments (POCUS and not using POCUS) were considered standard therapy and therefore no consent was deemed necessary. We, however, choose to use the same MET but asked to use POCUS only during even weeks and discouraged the use of POCUS during odd weeks. Because Zieleskiewicz et al. used two separate METs, theoretically the difference in the number of correct diagnosis was not only the result of the use of POCUS, but also due to differences between the achievements of the two separate teams although a large number of seniors and juniors randomly composed each MET. In our study, the MET physician was the same during both weeks (US+ and US−).

Furthermore, it is unclear in the Zielskiewicz et al. study who exactly performed the POCUS protocol, their METs are staffed by junior and senior physicians (with minimally a level 2 in thoracic ultrasound). We also found a significant impact on diagnostic accuracy, but in our study POCUS was done by residents only, this aspect is worth emphasizing; POCUS can be of an extra value to the less experienced physician during MET calls. We found that without POCUS the number of correct diagnosis was relatively low (51.1%). Although this has been found before in studies in which POCUS was done by more experienced staff [11, 20], we think that this low percentage can also be partially explained by the fact that in our study less experienced physicians (residents) included the patients.

Several studies show that training programs for residents in multi-organ POCUS have satisfactory results in terms of acquiring adequate ultrasound skills and increasing diagnostic abilities. [21–28]. Our study supports the evidence that residents may obtain clinically relevant POCUS skills in a relatively short period of time, including making the right diagnosis during a MET call. Our findings are of interest for other hospitals in which the MET is also staffed by residents; POCUS training will improve their diagnostic ability and certainty also during acute situations like MET deployments. Also important to stress is the fact that the use of the POCUS protocol never resulted in a change towards a completely incorrect diagnosis.

Because we asked the MET physician to note to a diagnosis before the use of POCUS and one after the use of POCUS, we could measure more precisely the effect of the use of POCUS and this was significant in the US+ group and also positive though not significant due to small numbers in the US− group. This also supports the fact that POCUS was the reason for more diagnostic accuracy.

The other study on the use of ultrasound by MET’s was published by Sen et al. [29]. In this small study of 50 patients on the effect on diagnosis of a combined lung and lower extremity vascular POCUS protocol was evaluated. They showed that lung POCUS was feasible, but due to the small number of patients there was no statistically significant effect on the number of correct diagnosis. They therefore concluded that the use of lung ultrasound was non-inferior to MET clinical assessment. It could well be that by the addition of cardiac POCUS and the inclusion of more patients Zieleskiewicz et al. and we were able to prove a beneficial effect of POCUS on diagnosis during MET calls.

We also evaluated the effect of POCUS on physician’s certainty. We showed that in the US+ and US− groups baseline certainty was quite high (eight on a 10 point VAS scale), but certainty increased significantly in the US+ group after the use of POCUS indicating that POCUS did not only improve certainty because certainty levels were low to begin with. There is one other study on the impact of POCUS on diagnostic certainty [5]. Shokoohi et al. looked at 118 ER patients and found that the use of a multi-organ POCUS protocol lowered uncertainty for a diagnosis. We are aware of the relatively small increase in certainty that was found in our study, but this finding could be psychologically important for residents during stressful clinical encounters such as MET calls. Junior physicians are less certain in the diagnostic process compared to more experienced colleagues [30].

We could not find differences in other outcome parameters (28-day mortality, initial treatment or supervisor attendance) possibly due to the small number of included patients. It will be difficult to design a study on the use of POCUS during MET calls large enough to detect differences in outcome parameters such as mortality. Preferably, a multicenter trial could help in including large enough numbers of patients. On the other hand, one could argue that the existing evidence is sufficient to support the incorporation of POCUS in MET protocols.

Our study has several limitations. This study was conducted in a single center and focused on ward patients with respiratory and or hemodynamic deterioration. Therefore, the presented results may not necessarily apply to other clinical settings. There were some differences in baseline characteristics between the US+ and US− groups. Significantly more patients in the US+ group had a history of myocardial infarction and heart failure. We have no reason to believe that these baseline differences had a significant impact on the study results. Our study design is prone to selection bias, but also has several advantages as discussed above. We decided to exclude the MET physicians without sufficient POCUS training from the trial. This resulted in a substantial number of non-included patients (87).

Our POCUS protocol consisted out of a combination of 5 basic cardiac questions and for the lung the Blue protocol was used which is consistent with current international POCUS practice. No further prespecified POCUS flow chart was used which makes our findings perhaps difficult to validate by others.

The diagnoses of the MET physicians were compared to the final diagnosis made by one blinded experienced intensive care consultant 2 weeks afterwards (independent expert) on the basis of a thorough chart review. This method remains challenging although often used in POCUS studies [12, 13]. In our study, only one independent expert reviewed the charts and was blinded to the ultrasound findings and diagnosis made by the attending MET physician. He had absolutely no other role in the study and because he is a member of the hospital mortality committee he is experienced in extracting official diagnoses from chart review.

## Conclusion

We found that the use of a thoracic (cardiac and lung) POCUS protocol during MET calls due to respiratory and or hemodynamic deterioration has significant positive impact on establishing the correct diagnosis and a small but significant impact on MET physician’s diagnostic certainty.

## Supplementary Information

**Additional file 1.** Figure of Modified Early Warning Score (MEWS).

## Data Availability

The datasets used and/or analyzed during the current study are available from the corresponding author on reasonable request.

## Abbreviations

POCUS: Point-of-care ultrasound
MET: Medical emergency team
US: Ultrasound
ER: Emergency room
ICU: Intensive care unit
MEWS: Modified Early Warning Score
IVC: Inferior vena cava
ARDS: Acute respiratory distress syndrome
AHF: Acute heart failure
AECOPD: Acute exacerbation of chronic obstructive pulmonary disease
CRF: Case research form
CPR: Cardiopulmonary resuscitation
N: Number
GI: Gastro-intestinal
AAD: Acute admission department
BMI: Body mass index
BP: Blood pressure
SD: Standard deviation
WBC: White blood cell count
COPD: Chronic obstructive pulmonary disease
ILD: Interstitial lung disease
VAS: Visual analogue scale
