# Correction to: A point-of-care thoracic ultrasound protocol for hospital medical emergency teams (METUS) improves diagnostic accuracy

**DOI:** 10.1186/s13089-021-00233-7

**Published:** 2021-06-16

**Authors:** M. J. Blans, E. Bousie, J. G. van der Hoeven, F. H. Bosch

**Affiliations:** 1 Department of Intensive Care, Rijnstate Hospital, PO box 9555, 6800 TA Arnhem, The Netherlands; 2 Department of Intensive Care, Medical Center, Radboud University, PO box 9101, 6500 HB Nijmegen, The Netherlands; 3 Department of Internal Medicine, Rijnstate Hospital, PO box 9555, 6800 TA Arnhem, The Netherlands

## Correction to: Ultrasound J (2021) 13:19 10.1186/s13089-021-00229-3

Following the publication of the original article [1], we were notified that the first and last name order in the authorship list has been swapped.

The original article has been corrected.
