# Supplementary figures and images for: A point-of-care thoracic ultrasound protocol for hospital medical emergency teams (METUS) improves diagnostic accuracy
Source: Ultrasound J. 2021 Jun 4;13:29. doi: 10.1186/s13089-021-00229-3 (PMC8178424; doi:10.1186/s13089-021-00229-3)

Additional file 1.


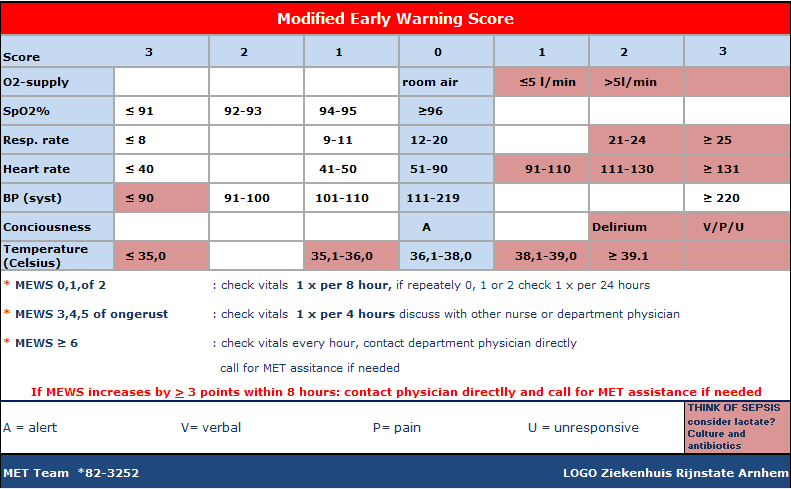


Figure of Modified Early Warning Score (MEWS)

Supplement: Supplementary file 1 — Additional file 1. Figure of Modified Early Warning Score (MEWS). [file 13089_2021_229_MOESM1_ESM.docx]
